# Kinetic study of uranium residue dissolution in ammonium carbonate media

**DOI:** 10.1007/s10967-014-3396-3

**Published:** 2014-08-24

**Authors:** B. Kweto, D. R. Groot, E. Stassen, J. Suthiram, J. R. Zeevaart

**Affiliations:** 1 Department of Materials Science & Metallurgical Engineering, University of Pretoria, Lynnwood Road, Pretoria, 0002 South Africa; 2 Applied Chemistry, South African Nuclear Energy Corporation (Necsa), Pelindaba, Pretoria, South Africa; 3 Department of Science and Technology, Preclinical Drug Development Platform, North West University, Potchefstroom, South Africa

**Keywords:** Ammonium carbonate, Hydrogen peroxide, Leaching, Chemical reaction, Extraction rate, Activation energy and uranium residue

## Abstract

The purpose of this study was to determine the kinetics of the dissolution of a uranium residue in ammonium carbonate media. The residue is generated in the production of medical isotopes. The effects of parameters, such as varying peroxide and carbonate concentrations, dissolution time as well as temperature on the extraction rate have been separately studied. Results indicate complete dissolution of the residue at 60 °C, after 30 min, in ammonium carbonate solution enriched with hydrogen peroxide. The yield and rate of uranium extraction were found to increase as a function of both temperature, in the range of 25–60 °C, and hydrogen peroxide concentration. The extraction process was governed by chemical reaction as the activation energy was found to be 45.5 kJ/mol. The order of reaction with respect to uranium concentration was found to be approximately first order.

## Introduction

The uranium residue consists of insoluble precipitate that forms when target plates of uranium and aluminum alloy are dissolved during the production of molybdenum-99 for use in technetium generators. This process was developed by the Karlsruhe Nuclear Research Center in Germany [1].

During this process, an alloy of enriched uranium and aluminum is irradiated in a thermal neutron flux. After short cooling periods, the plates are dissolved in a strong base medium of sodium hydroxide. This results in the dissolution of the aluminum matrix as well as the fission products: molybdenum, cesium, strontium, barium, antimony, tellurium, iodine and a portion of the ruthenium and zirconium. The insoluble residue that remains contains more than 90 % of uranium that is present in a mixture of oxidation states [2].

The uranium content in the residue was believed to be predominantly in a U(IV) oxidation state. Thus, understanding of the dissolution of the simpler pure uranium dioxide in carbonate solutions was essential for the understanding of the dissolution of the more complex residue. Solid–liquid ratio, type of oxidants and carbonate concentrations were varied to determine the rate laws governing the dissolution. The resulting kinetics model was then applied to the dissolution of the residue from the molybdenum process for uranium recovery.

Some studies on the dissolution kinetics of uranium dioxide particles have been conducted by different authors to determine the influence of reagent concentration, type of oxidants and temperature on the dissolution rate of uranium dioxide in alkaline solution.

Smith and colleagues reported that the dissolution rate of uranium dioxide powder increases linearly over the temperature range of 15–60 °C in 1 M ammonium carbonate with varied concentrations of hydrogen peroxide [3]. This has been confirmed by Pierce and colleagues who observed that the rate of uranium dioxide dissolution in carbonate solution increased by an order of magnitude with a 30 °C increase in temperature [4].

The rate of uranium dioxide dissolution under oxidizing conditions in carbonate/bicarbonate media was found to be directly proportional to the total hydrogen carbonate concentration by Grandstaff [5]. Generally, within the pH range from 8.3 to 10.3 the rate of dissolution of uranium dioxide is independent of carbonate/bicarbonate ratio because the carbonate and bicarbonate ions play equivalent roles; the stable uranyl tri-carbonate species being dominant throughout the range.

Literature suggests an order of 0.5 or 1 when using only dissolved oxygen as an oxidant, in the absence of hydrogen peroxide [6–8]. Hiskey reported that the order of carbonate in the dissolution of uranium dioxide in a sodium carbonate and hydrogen peroxide solutions is 1 [9].

Investigating the oxidative dissolution of uranium dioxide in alkaline media, Clarens et al. [10] observed that the dissolution rate of uranium dioxide increased with increasing hydrogen peroxide concentration. De Pablo et al. [7] found that the rate of uranium dioxide dissolution in the presence of sodium carbonate and hydrogen peroxide solutions was first order with respect to hydrogen peroxide.

However, there is a disagreement over the order of hydrogen peroxide while dissolution is done in ammonium carbonate. Smith and colleagues reported that the dissolution rate increased linearly with a slope of 2.41 with hydrogen peroxide concentration in 1 M ammonium carbonate for peroxide concentration between 0.05 and 2 M [2]. Hiskey, on the other hand, found that the order of hydrogen peroxide was 0.5 in 0.5 M ammonium carbonate for concentrations between 0.009 and 0.220 M [9].

The temperature dependence of the dissolution of uranium dioxide in carbonate and peroxide solutions has not been well studied. Casas et al. [11] reported the activation energy of uranium dioxide dissolution in sodium bicarbonate and hydrogen peroxide as 40 kJ/mol. The experiments have been performed in a temperature range from 20 to 50 °C. Hiskey also reported activation energies of 42.9 and 46.5 kJ/mol in ammonium carbonate at 1.0 and 7.9 atm of oxygen pressure respectively, in the same temperature range [6].

Thus, it can be seen that the concentration of the reagents and the temperature of the solution have an impact on the dissolution rate of uranium dioxide in carbonate media. However, the working temperature, while leaching with ammonium carbonate in an open beaker, must be kept below 60 °C as its solutions decompose to ammonia and carbon dioxide above this temperature [12].

An ammonium carbonate based dissolution process of the residue using hydrogen peroxide as an oxidant for uranium recovery has been developed and described in the previous paper [13]. Therefore, the present paper deals with the results obtained during the kinetics study of uranium dioxide dissolution and the residue dissolution in ammonium carbonate media.

## Materials and methods

### Uranium dioxide fuel pellets

Fuel pellets of uranium dioxide (99.9 % uranium dioxide) supplied by the South African Nuclear Energy Corporation (Necsa), were ground in a mortar prior to the start of the experiments. 70 g of the sample with a particle size of 38–106 µm was immersed for 48 h in a solution of 1 M ammonium carbonate, prepared with oxygen free water. The aim was to dissolve oxidized phases that may have formed on the surface of the pellets due to oxidation by air. The sample was then washed with water free of oxygen and leached.

### Simulated residue

100 g of simulated residue, with a particle size of 38–106 µm, was used. The material was supplied by Necsa and was not pre-treated for oxide layers removal prior to leaching. This was due to the fact that the sample was kept in a sealed container and was not exposed to atmospheric air.

### Experimental techniques

#### Experimental approach

The experimental approach involved first the dissolution of uranium dioxide samples in an open beaker or in the autoclave. Then, the resultant kinetics model was applied to the simulated residue samples.

Leaching variables considered were temperature, solid–liquid ratio and reagent concentration. Single variable testing was used for the optimization of the dissolution parameters. In this approach, a series of leaching tests were performed while changing only one variable at a time and maintaining the other variables at fixed values for a given set of measurements [14].

#### Open system

0.5 g of the uranium residue was transferred to a glass beaker containing ammonium carbonate solution and hydrogen peroxide of an appropriate concentration. The mixture was then heated to the required temperature with continuous stirring until the end of the experiment.

Solution pH was measured using a Metrohm 704 pH-meter with a combination pH electrode and temperature probe. The meter and electrode were calibrated using pH 7.00 and 10.00 buffer solutions.

After dissolution, the solution was allowed to cool to ambient temperature and then filtered through a weighed No 4 Whatman filter paper. The undissolved residue was collected, washed, dried for 24 h in the oven at 60 °C and then weighed to determine the amount of the undissolved sample.

Depending on the expected dissolved uranium, solutions collected were analyzed for uranium by use of the spectrophotometric technique.

#### Pressure leaching

The autoclave used for the dissolution of the sample was a Parr 4848 reactor controller that was connected to a stainless steel 4597 Micro Reactor with a 100 ml fixed head. The device has a working pressure up to 207 bar and maximum working temperature of 350 °C is permissible.

0.5 g of the simulated residue was added into ammonium carbonate and hydrogen peroxide solutions of an appropriate concentration. The mixture was then poured into the reaction vessel, which was sealed, and then heated to the required temperature with continuous stirring (500 rpm) until the end of the experiment. All experiments using ammonium carbonate solutions at 60 °C and above were performed in the leach autoclave as ammonium carbonate decomposes to ammonia and carbon dioxide above this temperature.

Sampling was not possible with this autoclave; therefore analysis was carried out after the experiment.

The final solution was filtered with Whatman No 4 filter paper and the residue obtained was washed, dried and weighed to determine the amount of the undissolved uranium.

The experimental conditions used for batch and autoclave leaching are shown in Table 1 below.

**Table 1:** Experimental conditions used for batch and autoclave leaching

Parameters	Batch leach	Autoclave leach
Temperature (°C)	25; 40; 50	60
Dissolution time (min)	30; 60; 90; 120; 240	30; 60; 180
Solid–liquid ratio	1:40; 1:60; 1:80	1:60; 1:80
Particles size (µm)	38–106	38–106
Agitation speed (rpm)	500	500
[(NH_4_)_2_CO_3_] (M)	0.1; 0.5; 1	0.1; 0.5; 1
[H_2_O_2_] (M)	0.1; 0.5; 1	0.1; 0.5; 1
Oxygen pressure (bar)		4
Total carbonate (M)	0.1; 0.5; 1	0.1; 0.5; 1

## Results and discussion

### Sample characterization

A sample was sent to Pelindaba Analytical Lab for X-ray fluorescence analysis in order to determine the major components present in the simulated residue. The results are indicated in Table 2.

**Table 2:** Semi-quantitative XRF results from analysis of the simulated residue

Determination	Results
Phosphorus	Trace
Silicon	Trace
Sodium	Minor
Uranium	Major
Aluminium	Minor

**Fig. 1:**
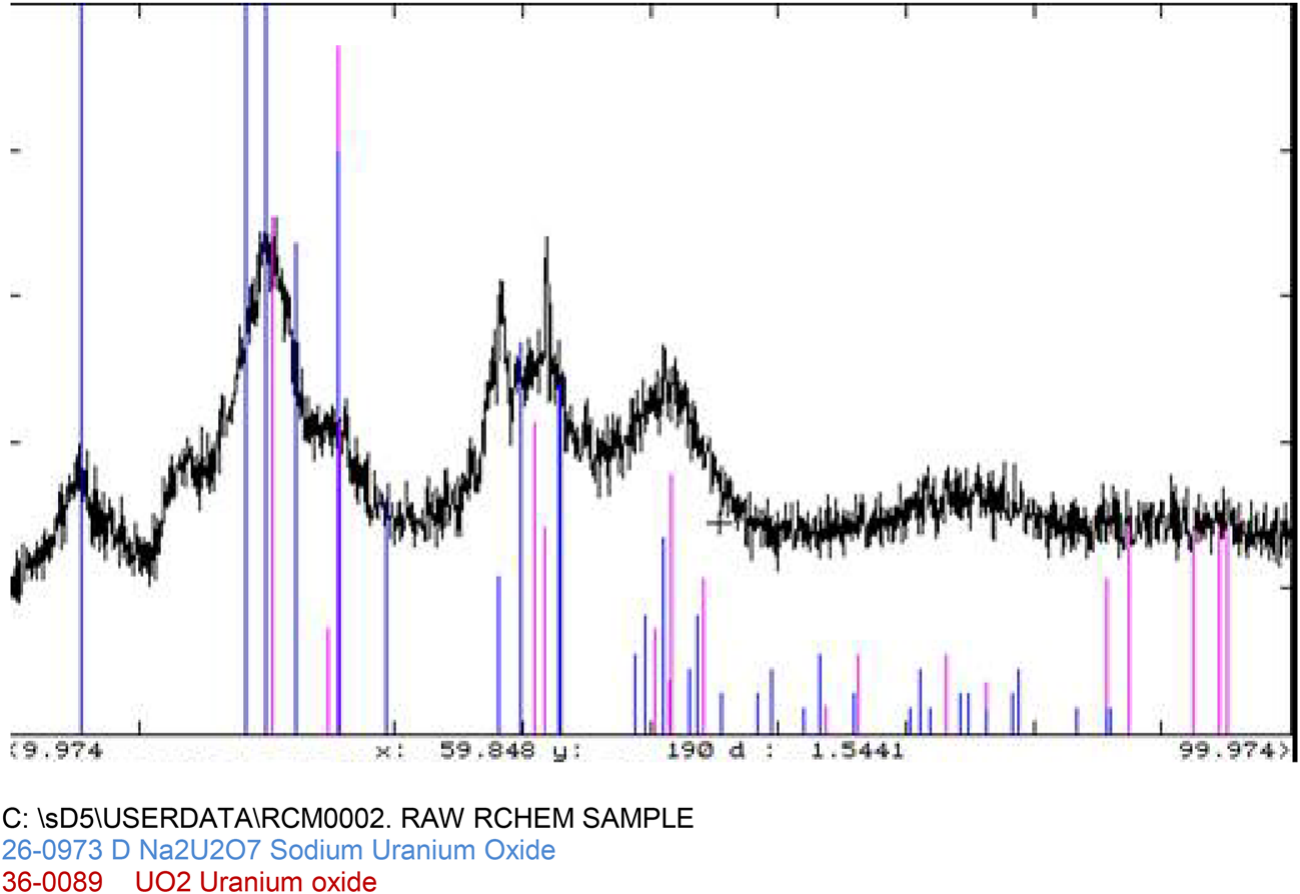
The diffractogram of the simulated residue

A second sample of the simulated residue was sent for X-ray diffraction in order to determine the oxidation state (see Fig. 1).

The diffractogram of the uranium sample (simulated) matched closely to that of uranium dioxide and Na_2_U_2_O_7_. These compounds of uranium were expected. The uranium is therefore present in a mixture of oxidation states [2].

### Uranium dioxide leach kinetics

#### Determining the rate law

The first step in understanding how a given chemical reaction occurs is to determine the form of the rate law. To decide whether the rate law for uranium dioxide dissolution in ammonium carbonate is first order, second order or zero order, an indication on whether the plot of ln[UO_2_], 1/[UO_2_] or [UO_2_] versus time is a straight line has to be found, respectively.

[UO_2_] represents the total concentration of uranium dissolved from the uranium dioxide sample.

**Fig. 2:**
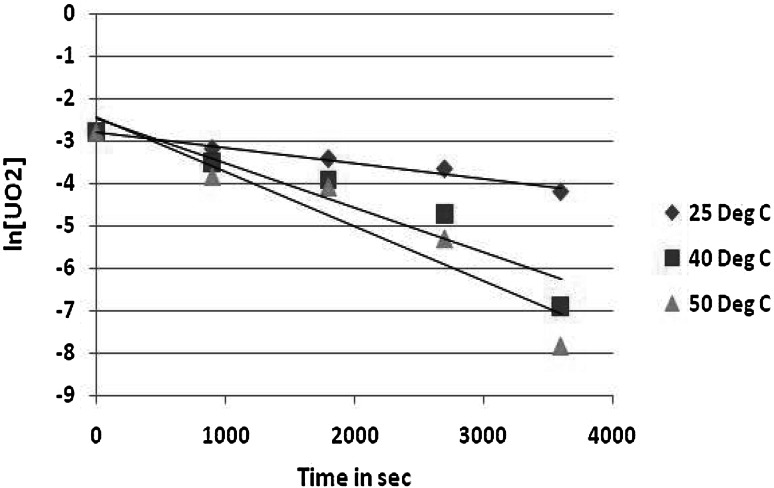
First order plot for uranium dioxide dissolution at different temperatures at 1 M (NH_4_)_2_CO_3_ and 1 M H_2_O_2_

From Fig. 2 below, it was found that the amount of uranium that dissolves over any experiment is small compared with the amount of uranium dioxide solid. This implies that the total solid surface area remains constant within any single test. Thus, the reaction rate is proportional to the exposed surface area of the crushed uranium dioxide pellets.

It was also observed that the regression line does fit the data in the plot ln[UO_2_] versus time at 25 °C. This confirms that the relationship between the two variables is certain enough to be useful. Thus the reaction is first order in UO_2_ under the reaction conditions used in this work. This observation is consistent with that reported by Sharma and colleagues who found the order of reaction to be unity while dissolving uranium dioxide in sodium carbonate-bicarbonate solution containing sodium hypochlorite as an oxidant [15].

#### Rate of decomposition of uranium dioxide

The chemical reaction of uranium dioxide dissolution in solutions of ammonium carbonate and hydrogen peroxide was studied using experiments in which the reactants were charged to a vessel and maintained at constant and uniform temperature.

The order of the rate law with respect to uranium dioxide concentration was verified by constructing a plot of ln [UO_2_] versus time. The value of the rate constant *k* was determined from the slope of the resultant line.

Since the reaction is first order in uranium dioxide, the slope of the line equals −*k*, where

$$ {\text{Slope}} = \frac{{\varDelta \left( {\ln \left[ {{\text{UO}}_{ 2} } \right]} \right)}}{\varDelta t} = - k. $$

Table 3 below show the rate obtained at different temperatures (°C) and solid–liquid ratio (g/ml).

**Table 3:** Values of *k* for different temperatures and solid–liquid ratio

*T* (°K)	*k*_1/40_ (s^−^)	*k*_1/60_ (s^−^)	*k*_1/80_ (s^−^)	RSD (%)
298	0.00035	0.00039	0.00043	10.2
313	0.00108	0.00114	0.00109	2.90
323	0.00132	0.00140	0.00120	7.70

The rate more than doubles going from 25 to 40 °C but *k* only increases slightly from the 40° to 50°. This fall-off in slope with increasing temperature may be due to catalytic decomposition of hydrogen peroxide to water and oxygen, as bubbling was observed.

For the solid liquid ratios, *k* is at its highest at a ratio of 1:60 for both 40° and 50° runs, decreasing slightly once the ratio is increased to 1:80. Thus, leaching at solid–liquid ratio of 1:60 appears to be more advantageous.

#### Activation energy for uranium dioxide dissolution

In order to obtain the value of the activation energy, ln *k* was plotted against (1,000/*T*), which resulted in a straight line for different solid–liquid ratios used. The values of activation energy and *R* ^2^ obtained for each solid–liquid ratio are shown in Table 4 below.

**Table 4:** Values of activation energy and *R* square at different solid–liquid ratio

S/L ratio (g/ml)	*E*_a_ (kJ)	*R* ^2^
1/40	44.2	0.94
1/60	42.5	0.94
1/80	33.9	0.89

It may be seen from these results that at solid–liquid ratios of 1:40 and 1:60 similar activation energies and regression coefficients were obtained with a standard deviation of 5.5 %.

The activation energy appears to decrease with the increase of solid–liquid ratio moving towards the diffusion controlled region. This is due to the fact that in the more diluted solution, the reaction is likely to be influenced by the reagents concentration then by the temperature used under the conditions investigated.

The activation energy was calculated using the following equation:

$$ E_{\text{a}} = - R\left[ {{\raise0.7ex\hbox{${\varDelta \ln \;k}$} \!\mathord{\left/ {\vphantom {{\varDelta \ln \;k} {\varDelta \left( \frac{1}{T} \right)}}}\right.\kern-0pt} \!\lower0.7ex\hbox{${\varDelta \left( \frac{1}{T} \right)}$}}} \right], $$

where *E*_a_ is the activation energy (J/mol), *R* is the gas constant (8.314 J/(K mol)), *T* is the leaching temperature (degree Kelvin) and *k* is the rate constant (*s* ^−^).

The average activation energy was found to be 40.2 kJ/mol (standard error 0.07), which is lower than what was reported in literature as being 57 and 51.1 kJ/mol by Sharma and du Preez [15, 16]. This could be due to the fact that hydrogen peroxide was used as the oxidant as opposed to sodium hypochlorite in the referenced studies.

The above activation energy of 40.2 kJ/mol is, therefore, in agreement with an activation controlled process.

**Fig. 3:**
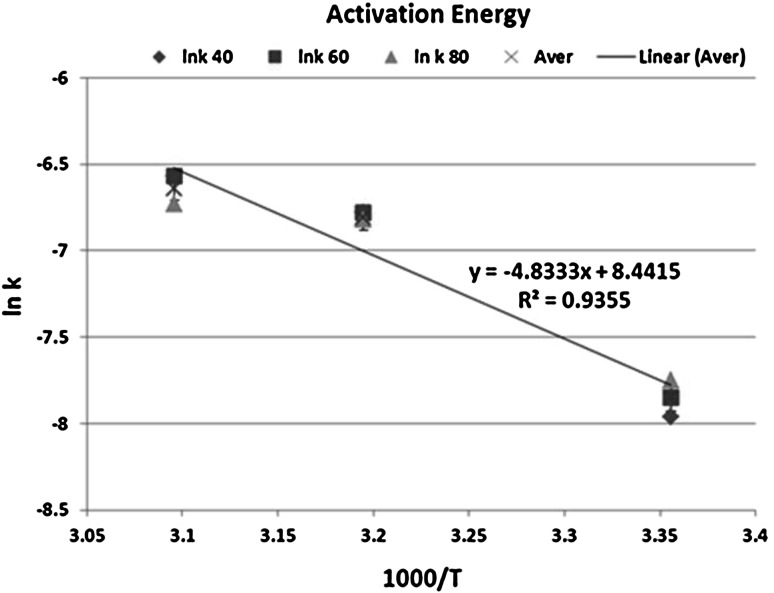
Plot of average ln *k* versus 1,000/*T* for uranium dioxide dissolution

The temperature dependency of dissolution reactions of uranium dioxide with different solid–liquid ratio characterized by the Arrhenius equation is further shown by Fig. 3. Thus we see that a plot of average ln *k* versus 1,000/*T* gives a straight line (error bars shown as 1.3 % of average).

The correlation coefficient *R* ^2^ for this plot is 0.93 and indicates that there is strong relationship between the rate of uranium dioxide dissolution and the temperature. Thus, as stated above, an increase of reaction temperature is expected to significantly increase uranium dioxide dissolution in the range of conditions investigated.

#### Order of reaction for hydrogen peroxide and ammonium carbonate

**Fig. 4:**
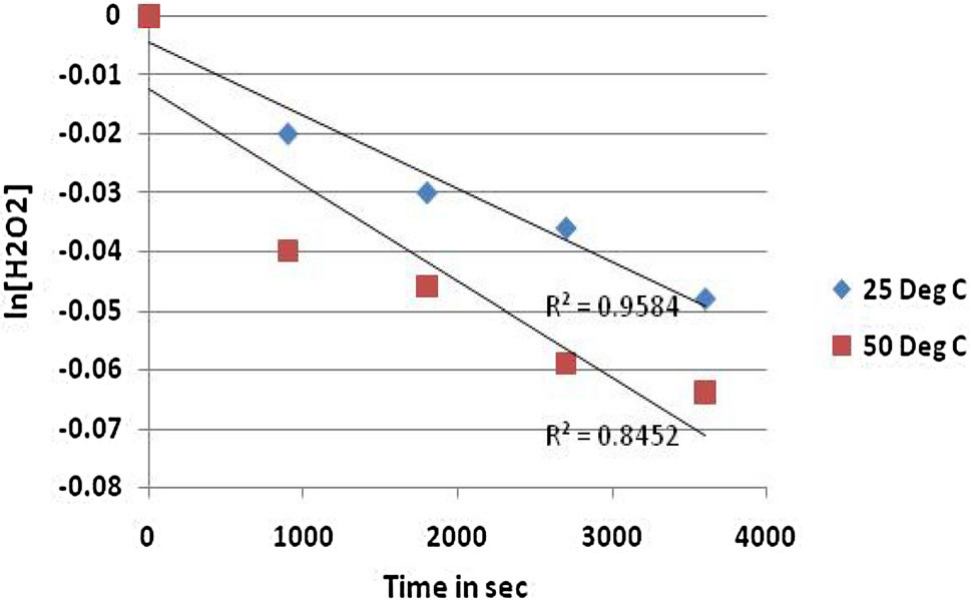
First order plot with respect to H_2_O_2_ at 25 and 50 °C

**Fig. 5:**
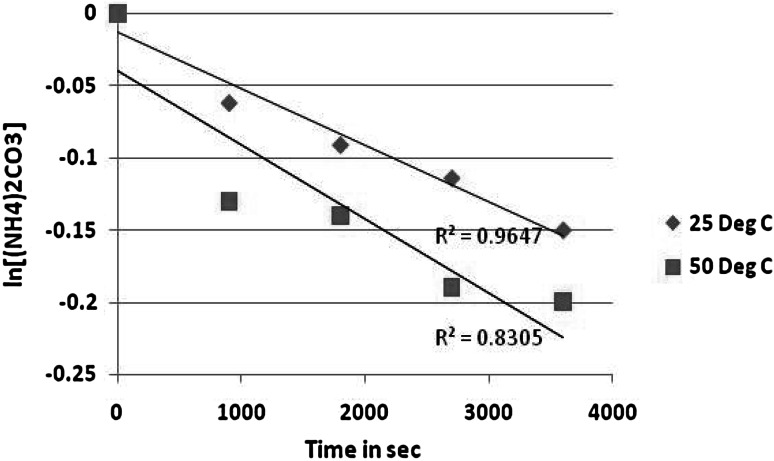
First order plot with respect to (NH_4_)_2_CO_3_ at 25 and 50 °C

The orders of reaction with respect to hydrogen peroxide (Fig. 4) and to ammonium carbonate (Fig. 5) were each found to be first order at 25 °C. This is also the case for 50 °C where a pseudo-first order reaction with respect to hydrogen peroxide and to ammonium carbonate was found but the fit was poorer.

### Uranium residue leach kinetics

#### Rate law for dissolution

In the dissolution studies of the simulated residue, it was found that uranium (VI) contained in the residue leaches quickly under non-oxidizing conditions. Thus, the kinetics study of the residue was done taking into consideration only the amount of the dissolved uranium (IV).

The simulated residue was first leached in 1 M ammonium carbonate solution then the solution was filtered. The undissolved residue was collected, washed then leached under oxidizing conditions for U(IV) dissolution. The aim was to check if the reaction orders are the same as what was obtained for pure uranium dioxide, ammonium carbonate and hydrogen peroxide.

It was observed that the data are well-correlated in the plot of ln[U(IV)] versus time (Figures not shown here). This implies that the dissolution of uranium residue is first order under the conditions investigated. First order reactions with respect to hydrogen peroxide and ammonium carbonate, respectively, were also observed.

#### Activation energy for uranium residue dissolution

From the values in Table 5, the rate constant increases as the temperature increases from 25 to 60 °C.

**Table 5:** Values of *k* for dissolution of the simulated residue at various temperatures

*T* (°K)	1,000/*T*	ln *k*
298	3.356	−8.3
323	3.096	−6.96
333	3.003	−6.93

**Fig. 6:**
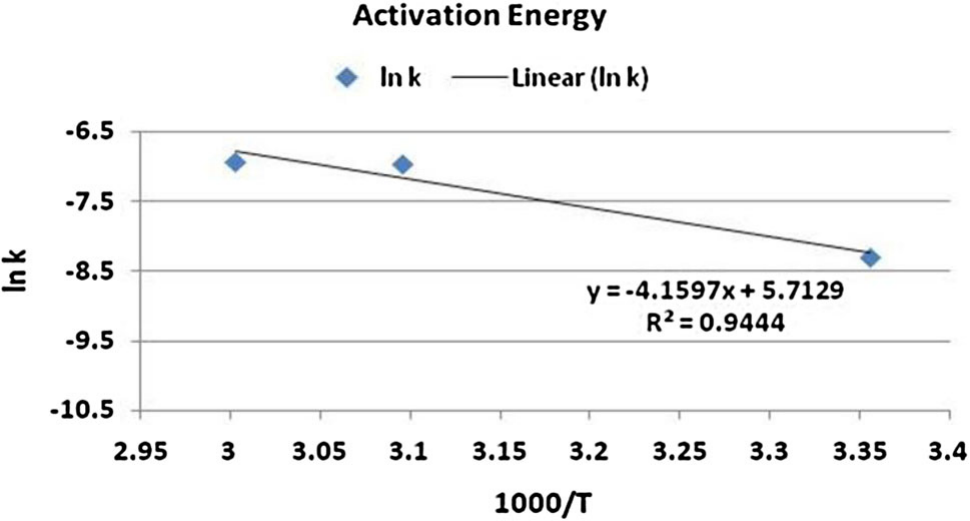
Plot of ln *k* against 1,000/*T* for uranium residue dissolution

It can be seen, from Fig. 6, that the kinetics of dissolution of uranium (IV) in the simulated residue shows a similar temperature dependence to that observed for pure uranium dioxide. The activation energy was found to be 45.5 kJ/mol (activation controlled process), which is above the activation energy found for pure uranium dioxide dissolution (40.2 kJ/mol) under the same experimental conditions. This is due to the fact that initial leaching of the pure uranium dioxide is quite rapid compared to the leaching of uranium (IV) contained in the simulated residue.

## Conclusions

This study has enabled determination of leaching kinetics for various leaching conditions of uranium residue in carbonate media containing hydrogen peroxide oxidant.

The activation energy for the dissolution of simulated uranium residue was found to be 45.5 kJ/mol and confirms a chemically controlled process for uranium residue dissolution in ammonium carbonate solution with hydrogen peroxide under the conditions investigated. The order of reaction with respect to uranium concentration, ammonium carbonate solution and hydrogen peroxide concentration each was found to be approximately first order.

The kinetics of dissolution of the uranium in the simulated residue was found to be similar to that of uranium dioxide dissolution in the presence of hydrogen peroxide, but with slightly higher activation energy.
